# Mutual information density of stochastic integrate-and-fire models

**DOI:** 10.1186/1471-2202-14-S1-P245

**Published:** 2013-07-08

**Authors:** Davide Bernardi, Benjamin Lindner

**Affiliations:** 1 Bernstein Center for Computational Neuroscience, Berlin 10115, Germany; 2 Department of Physics, Freie Universität Berlin, Berlin, Berlin 14195, Germany; 3 Department of Physics, Humboldt-Universität zu Berlin, Berlin, Berlin 12489, Germany

## 

The coherence function of integrate-and-fire neurons shows low-pass properties in the most diverse firing regimes [1]. While the coherence function provides a good approximation to the full information transfer properties in the case of a weak input, for a strong input non-linear encoding could play an important role. The complete information transfer is quantified by Shannon's mutual information rate [2] which has been estimated in certain biological model systems [3]. In general, the exact analytical calculation of the mutual information rate is unfeasible and even the numerical estimation is demanding [4].

Numerical calculation of the mutual information rate is now a commonly adopted practice, but it does not indicate what aspects of the stimulus are best represented by the neuronal response. We developed a numerical procedure to directly calculate a frequency-selective version of the mutual information rate. This can be used to study how different frequency components of a Gaussian stimulus are encoded in neural models without invoking a weak-signal paradigm.
